# Authors’ Reply: Clarifying Blood Indices in Patients With Ovarian Cancer

**DOI:** 10.2196/74931

**Published:** 2025-08-13

**Authors:** Jiahua Zhang

**Affiliations:** 1 Ward 2, Gynaecological Oncology Centre, Peking Union Medical College Hospital, Chinese Academy of Medical Sciences, No.1 Shuaifuyuan, Dongcheng District, Beijing, 100730, China, 86 13582506099

**Keywords:** WeChat, nutrition management, ovarian cancer, chemotherapy, mobile health

We agreed with the authors of the letter to the editor [1] that Figure 7 simply describes “the changes in blood cells.” Our study [2] only focused on the effect of nutritional status on the inflammatory response of the patients but neglected an important point: the changes in blood cells such as “leukocytes, lymphocytes, neutrophils, and platelets” were also closely related to the occurrence of bone marrow suppression (BMS) in the patients [3]. Figure 7 illustrates that the nutritional intervention program in this study can reduce the occurrence of BMS in patients to a certain extent.

We couldn’t agree more with the letter’s suggestion. We would like to include the benefits of improved BMS in the Discussion section of our study, which would primarily include the following:

- Improvement in BMS can contribute to the patient’s quality of life during chemotherapy [4].
- Reversal of the hematocrit condition corresponds to a reduction in the number or degree of the patient’s chemotherapy symptoms: elevated neutrophils lead to lower levels of fatigue, fewer persistent or chronic infections, and less nausea/vomiting [5].
- An increase in hemoglobin improved the patient’s appetite and reduced vertigo [6].
- An increase in the number of platelets reduced the incidence of hemorrhage [7].

## Abbreviations

BMS: bone marrow suppression
